# Brain grey and white matter predictors of verbal ability traits in older age: The Lothian Birth Cohort 1936

**DOI:** 10.1016/j.neuroimage.2017.05.052

**Published:** 2017-08-01

**Authors:** Paul Hoffman, Simon R. Cox, Dominika Dykiert, Susana Muñoz Maniega, Maria C. Valdés Hernández, Mark E. Bastin, Joanna M. Wardlaw, Ian J. Deary

**Affiliations:** a Centre for Cognitive Ageing and Cognitive Epidemiology, University of Edinburgh, UK; b Department of Psychology, University of Edinburgh, UK; c Scottish Imaging Network, a Platform for Scientific Excellence (SINAPSE) Collaboration, Edinburgh, UK; d Brain Research Imaging Centre, Neuroimaging Sciences, Centre for Clinical Brain Sciences, University of Edinburgh, UK

**Keywords:** Semantic knowledge, Anterior temporal lobe, Speech production, Individual differences

## Abstract

Cerebral grey and white matter MRI parameters are related to general intelligence and some specific cognitive abilities. Less is known about how structural brain measures relate specifically to verbal processing abilities. We used multi-modal structural MRI to investigate the grey matter (GM) and white matter (WM) correlates of verbal ability in 556 healthy older adults (mean age = 72.68 years, s.d. = .72 years). Structural equation modelling was used to decompose verbal performance into two latent factors: a storage factor that indexed participants’ ability to store representations of verbal knowledge and an executive factor that measured their ability to regulate their access to this information in a flexible and task-appropriate manner. GM volumes and WM fractional anisotropy (FA) for components of the language/semantic network were used as predictors of these verbal ability factors. Volume of the ventral temporal cortices predicted participants’ storage scores (β = .12, FDR-adjusted *p* = .04), consistent with the theory that this region acts as a key substrate of semantic knowledge. This effect was mediated by childhood IQ, suggesting a lifelong association between ventral temporal volume and verbal knowledge, rather than an effect of cognitive decline in later life. Executive ability was predicted by FA fractional anisotropy of the arcuate fasciculus (β = .19, FDR-adjusted *p* = .001), a major language-related tract implicated in speech production. This result suggests that this tract plays a role in the controlled retrieval of word knowledge during speech. At a more general level, these data highlight a basic distinction between information representation, which relies on the accumulation of tissue in specialised GM regions, and executive control, which depends on long-range WM pathways for efficient communication across distributed cortical networks.

## Introduction

In humans, the characteristics of both grey and white matter brain structures are informative predictors of the level and age-related change in cognitive abilities (e.g., Deary et al., 2010b; Ritchie et al., 2015). Higher general intelligence has been associated with greater brain volume and cortical thickness in a wide network of grey matter (GM) regions, principally in frontal and parietal cortices (Deary et al., 2010b, Jung and Haier, 2007). The structure of white matter (WM) tracts also makes a contribution to cognitive abilities (Ziegler et al., 2010). In older adults general fluid-type intelligence was associated moderately with a global measure of fractional anisotropy (FA) in WM tracts across the brain (Penke et al., 2012). This association was mediated entirely by a latent trait of information processing speed, suggesting that the efficiency of long-range neural connections contributes to ensuring efficient communication between brain regions, which in turn benefits complex cognitive functions.

Much of research linking brain structural indices with cognitive functions has focused on fluid-type cognitive abilities (Horn and Cattell, 1967), such as reasoning, working memory, executive function, and processing speed. Less is known about the aspects of brain structure that predict performance on verbal tasks that probe knowledge of words and their meanings. Such tasks are typically thought to depend heavily on crystallised-type abilities – i.e., stored knowledge. Colom et al. (2009) found that a measure of crystallised intelligence, based on tests of vocabulary, and verbal and numerical reasoning, was associated with greater GM volumes in many of the frontal and parietal sites linked to fluid abilities. In addition, however, crystallised ability was uniquely linked with greater volume in the anterior temporal cortex. This finding has been replicated by others (Choi et al., 2008), and is congruent with the suggestion that anterior temporal regions play a key role in representing semantic knowledge (Binder and Desai, 2011, Hoffman et al., 2014, Patterson et al., 2007). Semantic knowledge refers to our store of word and object concepts and thus is involved in most verbal tasks.

Few studies have focused specifically on the structural neural correlates of semantic knowledge in healthy individuals. de Zubicaray et al. (2011) found that semantic ability in older adults, measured by extracting the first principal component from a range of verbal and non-verbal semantic tasks, was correlated with GM volumes in the anterior temporal cortex. In this study, better performance was associated with *reduced* GM volume. In the same study, higher semantic scores were linked to greater FA in the uncinate fasciculus and inferior fronto-occipital fasciculus, two tracts which link temporal regions with prefrontal cortex. Another study failed to find any GM or WM regions that were linked specifically with performance on verbal semantic tasks (Ziegler et al., 2010). In both of these studies, however, sample size was relatively modest (*N*=55 in de Zubicary et al.; *N*=38 in Ziegler et al.). The number of participants in the present study (*N*=556) is an order of magnitude greater than these previous investigations.

Although few studies have investigated how the structure of the healthy brain is associated with semantic abilities, a rich neuropsychological literature has linked impairments in semantic processing with distinct areas of brain damage. The syndrome of semantic dementia (also known as the semantic variant of primary progressive aphasia) is characterised by a selective and often profound deterioration in semantic knowledge, accompanied by atrophy to anterior temporal regions (Hodges and Patterson, 2007). In this condition, the amount of cortical atrophy in the anterior fusiform gyri is strongly predictive of the severity of patients’ semantic impairment, suggesting a major role for this region in representation of semantic knowledge (Butler et al., 2009, Mion et al., 2010). Semantic dementia is also associated with damage to WM tracts connecting the temporal cortex to other sites, including the uncinate, arcuate and inferior longitudinal fasciculi (Acosta-Cabronero et al., 2011, Agosta et al., 2010). It is not clear at present, however, how WM damage contributes to the loss of semantic knowledge in this condition.

Impairments in semantic processing also occur as a consequence of damage to prefrontal and posterior temporoparietal cortex in stroke (Berthier, 2001, Noonan et al., 2010). However, whereas semantic dementia patients suffer from degradation of semantic representations, patients with prefrontal and temporoparietal damage have intact knowledge representations but fail to access and use these appropriately (Jefferies, 2013, Jefferies and Lambon Ralph, 2006). This neuropsychological dissociation is consistent with current theories which hold that semantic abilities are underpinned by two interacting but distinct systems: a store of semantic knowledge and an executive system that regulates flexible and goal-directed access to that information (Hoffman et al., 2015, Rogers et al., 2015). The executive element of semantic processing is critical because we hold a wide range of information about particular words/concepts and situations often require us to select specific aspects of this information while inhibiting others (e.g., selecting the contextually appropriate interpretation of words with multiple meanings; Hoffman et al., 2011; Rodd et al., 2005). Functional neuroimaging studies have implicated left inferior prefrontal, posterior middle temporal, and inferior parietal regions in these executive processes (Noonan et al., 2013). Less is known about potential WM contributions to executive semantic processing, though it has been suggested that the uncinate fasciculus may play an important role (Harvey et al., 2013). This tract connects temporal and frontal cortices.

**Fig. 1:**
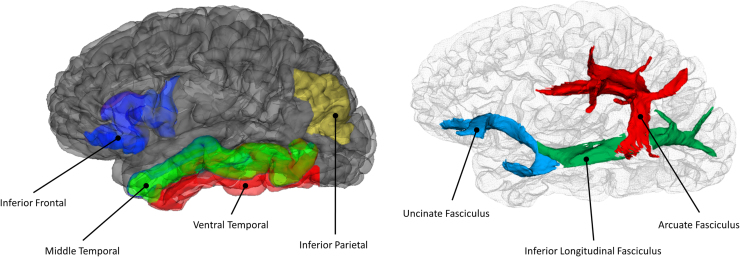
A schematic illustration of regions and tracts implicated in verbal-semantic processing. Cortical regions of interest are displayed on the cerebral mantle (left), and white matter tracts of interest are shown through a glass brain (right).

These findings in clinical populations suggest that verbal ability is underpinned by a store of verbal-semantic representations and by executive processes involved in accessing them. They also suggest that these two elements have distinct neural correlates. Despite this, no studies have investigated whether individual differences in these abilities in healthy individuals can be predicted by brain structure. In the present study, we investigated GM and WM associations with verbal abilities in the Lothian Birth Cohort 1936 (LBC1936; Deary et al., 2012; Deary et al., 2007), a longitudinal study of cognitive ageing that includes structural neuroimaging data for over 700 healthy older adults. We used structural equation modelling to isolate a factor of verbal ability associated with storage of verbal knowledge and one indexing executive processes that govern access to that knowledge. We then used multi-modal MRI to assess associations of key GM and WM structures with both of these components. Four GM regions and three WM tracts were identified as being of potential importance, based on existing theories of verbal processing (shown in Fig. 1). Their volumes/FA were used as predictors of verbal abilities. The Method section contains more details of the regions and tracts and why they were chosen *a priori* as predictors. We hypothesised that storage of verbal knowledge would be predicted by volume in GM regions implicated in semantic processing, particularly regions of the ventral temporal lobes associated with representation of semantic information. We expected WM tract FA to be associated with the executive component of verbal ability, since regulating access to information requires the co-ordination of activity across distributed cortical sites.

## Method

### Participants

Participants were members of the Lothian Birth Cohort (LBC1936; Deary et al., 2007), a sample of older adults residing in the Lothian area of Scotland, most of whom took part, at approximately 11 years of age, in the 1947 Scottish Mental Survey (Scottish Council for Research in Education, 1949). This survey involved completion of the Moray House Test No. 12 (Deary et al., 2007), which provides a measure of general cognitive ability in childhood. Subsequently, they have taken part in multiple waves of assessment in later life. The present data are taken from the second wave in older age, at which 732 individuals underwent T1-weighted structural and diffusion tensor MRI in addition to completing a battery of cognitive tests and other procedures (Deary et al., 2012, Wardlaw et al., 2011). Their mean age at assessment was 72.68 years (s.d.=.72). All participants provided written informed consent before testing. The LBC1936 study was approved by the Multi-Centre Research Ethics Committee for Scotland (MREC/01/0/56), the Lothian Research Ethics Committee (LREC/2003/2/29) and the Scotland A Research Ethics Committee (07/MRE00/58).

Six hundred and eighty participants provided neuroimaging data of suitable quality to determine GM volumes and FA tract data. Of these, 124 were excluded due to self-reported history of stroke or neurodegenerative disease, or evidence of stroke on MRI scan. The final sample therefore consisted of 556 participants (261 female). Mean demographic and cognitive data for the sample are provided in Table 1.

**Table 1:** Demographic and cognitive summary data for the sample.

	Mean (range)	s.d.
Sex	52% M: 48% F	
Handedness	5% L: 95% R	
Age	72.5 (71 – 74)	.7
Years of education	10.8 (9 – 14)	1.1
WAIS III subtests		
Symbol search	24.9 (3 – 43)	6.1
Digit-symbol coding	57.1 (22 – 94)	12.0
Matrix reasoning	13.2 (4 – 25)	4.8
Letter-number sequencing	11.0 (1 – 20)	3.1
Digit span backwards	7.9 (2 – 14)	2.3
Block design	33.6 (11 – 65)	9.9

### Cognitive assessments

Participants completed two tests of irregular word reading: the National Adult Reading Test (NART; Nelson and Willison, 1991) and the Wechsler Test of Adult Reading (WTAR; Holdnack, 2001). Both tests require participants to read aloud a series of words whose pronunciations do not conform to typical letter-sound mappings or stress patterns (e.g., *aisle*). Such words tend only to be read correctly if participants have prior knowledge of them; thus these tests provide indication of the breadth of stored verbal knowledge available to each participant.

Participants also completed phonemic verbal fluency tasks, in which they were asked to produce as many words as possible in one minute (each) beginning with the letters C, F and L. They were instructed not to produce proper nouns, to repeat words or to produce the same word with different endings. Verbal fluency draws on a participant's store of verbal knowledge but additionally places high demands on executive processes governing search and selection of appropriate lexical candidates (Lezak, 2004, MacPherson et al., 2015, Rogers et al., 2015). In contrast, the reading tasks place minimal demands on executive processes because the appropriate lexical-semantic targets are fully specified by the stimulus. Although there are other forms of fluency task that place even greater demands on executive ability (e.g., alternation between two categories or words with a specific number of letters), an advantage of the phonemic fluency task is that it has simple instructions that can be understood by all participants.

### Neuroimaging measures

Participants underwent whole brain structural and diffusion tensor MRI (DTI), described in detail elsewhere (Wardlaw et al., 2011). Structural MRI included T2- (T2W), T2*-(T2*W) and FLAIR-weighted axial sequences, and a high resolution 3D T1-weighted volume scan on a GE Signa Horizon HDxt 1.5 T clinical scanner (General Electric, Milwaukee, USA) operating in research mode using a self-shielding gradient set (maximum gradient 33 mT/m), and an 8-channel phased-array head coil. DTI consisted of high angular resolution 2 mm isotropic voxel diffusion MRI (seven T2- and 64 diffusion-weighted (b=1000 s/mm2) axial single-shot spin-echo echo-planar imaging volumes). All structural MRI data were examined by a consultant neuroradiologist (JMW) to exclude any participants with evidence of previous infarcts or lacunae. The rate and nature of incidental findings in this cohort has previously been reported in detail (Sandeman et al., 2013).

Intracranial volume (ICV) was measured for each participant by combining several sequences (T1-, T2-, T2* and FLAIR-weighted) and applying a validated semi-automated multi-spectral fusion technique (Valdés Hernández et al., 2010) whose output was visually examined for accuracy (Wang et al., 2012). Cortical surface reconstruction and regional parcellation was conducted using FreeSurfer v5.3 (https://surfer.nmr.mgh.harvard.edu) according to the Desikan atlas (Desikan et al., 2006). Briefly, each T1-W volume was processed according to previously-described steps (Fischl and Dale, 2000, Fischl et al., 2001, Fischl et al., 1999a, Fischl et al., 1999b, Fischl et al., 2004, Ségonne et al., 2004, Ségonne et al., 2007, Sled et al., 1998) as follows: removal of non-brain tissue, intensity normalisation, tessellation of tissue boundaries, automated topology correction, followed by inflation and registration of the cortical surfaces to a spherical atlas (using folding patterns to match cortical geometry). Following visual quality control (removing those with tissue identification and boundary positioning errors), we computed the volumes of four regions of interest, shown in Fig. 1, that have been reliably implicated in verbal semantic processing in the functional neuroimaging and neuropsychological literature:

### Inferior frontal gyrus (IFG)

IFG plays an important role in selection and regulation of verbal information (Thompson-Schill et al., 1997). We computed its volume by summing the volumes of pars orbitalis, pars triangularis and pars opercularis.

### Ventral temporal (VT) lobe

The ventral surface of the anterior temporal lobes is highly active during verbal semantic processing and is thought to support a store of semantic representations (Binney et al., 2010). We defined this region as the sum of the volumes of the inferior temporal and fusiform gyri. The Desikan atlas makes no distinction between anterior and posterior areas of the temporal gyri, so this region included anterior regions linked with semantic processing as well as posterior occipitotemporal regions more closely associated with visual processing.

### Middle temporal gyrus (MTG)

Posterior MTG is a key node in the language processing network and has been linked with lexical access and executive control (Dronkers et al., 2004). Functional activity in this region also predicts semantic ability in young adults (Wei et al., 2012). We used the volume of MTG as defined in the Desikan atlas (2006), which covers the full length of the gyrus.

### Inferior parietal cortex (IPC)

IPC is frequently activated in neuroimaging studies of language processing and may be involved in either representation of semantic information (Binder and Desai, 2011) or in executive regulation (Noonan et al., 2013). The Desikan atlas (2006) definition of this region includes the angular gyrus and the lower bank of intraparietal sulcus.

For each region of interest, volumes were calculated for the left and right hemispheres separately. As left and right volumes were highly correlated in every case (see Supplementary Table 1), they were averaged to give a single set of values for each participant, which were entered into the analyses described below. Similar results were obtained when analyses were repeated using left hemisphere values only.

Diffusion MRI data were preprocessed using FSL tools (FMRIB, Oxford, UK; http://www.fmrib.ox.ac.uk) which extracted the brain, eliminated bulk patient motion and eddy current-induced artefacts, and estimated FA in each brain voxel (Pierpaoli et al., 1996). Connectivity data were generated using BedpostX/ProbTrackX, with a two-fiber model and 5000 streamlines to reconstruct tracts of interest (Behrens et al., 2007). Twelve tracts were extracted using probabilistic neighbourhood tractography implemented in the TractoR package (Clayden et al., 2011; http://www.tractor-mri.org.uk). This method of tractography has good reproducibility (Clayden et al., 2009). Tract masks obtained through probabilistic neighbourhood tractography were overlaid on the FA parametric maps and tract-averaged FA values, weighted by the connection probability, were determined for each tract in every subject. FA values for three tracts of interest were used in the present study. As FA values in the left and right hemispheres were strongly correlated (see Supplementary Table 1), we again averaged across hemispheres (though using left hemisphere values only produced similar results).

### Uncinate fasciculus (Unc FA)

The uncinate fasciculus is a major tract that connects the anterior temporal lobes with ventral prefrontal cortex. As both of these regions are involved in semantic processing, it has been hypothesised that this tract contributes to language processing as part of the ventral language route (Harvey et al., 2013, Parker et al., 2005). However, the necessity of this tract for language processing has been questioned by others (Duffau et al., 2009).

### Arcuate fasciculus (Arc FA)

The arcuate fasciculus connects auditory-phonological regions in the posterior superior temporal lobe with prefrontal and premotor regions involved in speech production. This tract is the major element of the dorsal language route and is believed to play a critical role in language tasks, particularly those that require co-ordination of phonological and motor processing, such as repetition (Parker et al., 2005, Saur et al., 2008).

### Inferior longitudinal fasciculus (ILF FA)

The ILF links anterior temporal cortex with occipital regions. It may play a role in language comprehension and in particular in the accessing of semantic information from visual inputs, though its precise function is not clear (Bajada et al., 2015, Mandonnet et al., 2007).

### Statistical analysis

We assessed the relationships between verbal abilities and GM volumes and WM FA, using structural equation modelling. This extends our previous analysis of cognitive networks using multimodal structural MRI of putative network components (e.g., Cox et al., 2015). Analyses were performed using the lavaan package in R (Rosseel, 2012) and employed maximum likelihood estimation. Missing data were assumed to be missing at random (see Table 2 for incidences). Before being included in models, each variable was adjusted for sex and age at testing by obtaining residuals after regressing on these covariates. In addition, GM volumes were regressed on total ICV to adjust for head size.

**Table 2:** Descriptive statistics and correlation matrix for all measures.

	N	Mean (s.d.)	1	2	3	4	5	6	7	8	9	10	11	12	13	14	15
1. Age	556	72.5 (.7)	–														
2. ICV (mm^3^)	556	1453126 (142405)	−.03	–													
3. Age 11 IQ	525	101.3 (15.1)	−.07	.07	–												
4. Educational level	555	1.8 (1.3)	−.04	.17***	.51***	–											
5. NART	554	34.7 (8.1)	−.09*	.10*	.70***	.61***	–										
6. WTAR	554	41.4 (6.9)	−.07	.12**	.69***	.58***	.90***	–									
7. VF-C	555	15.2 (5.0)	−.09*	.08	.36***	.31***	.41***	.40***	–								
8. VF-F	555	14.6 (4.6)	−.05	.08	.36***	.30***	.40***	.39***	.73***	–							
9. VF-L	555	14.0 (4.6)	−.03	.09*	.34***	.27***	.40***	.42***	.70***	.72***	–						
10. IFG volume	527	8917 (1018)	−.05	.57***	.05	.10*	.09*	.11*	.03	.06	.07	–					
11. VT volume	528	17814 (2273)	−.09*	.61***	.21***	.22***	.18***	.21***	.08	.10*	.13**	.53***	–				
12. MTG volume	528	9569 (1266)	−.06	.59***	.15**	.19***	.15**	.20***	.05	.05	.06	.54***	.72***	–			
13. IPC volume	528	12061 (1519)	−.05	.54***	.12**	.12**	.13**	.17***	.03	.04	.02	.51***	.64***	.65***	–		
14. Arcuate FA	531	.44 (.04)	−.02	.04	−.01	−.06	.00	−.02	.06	.10*	.10*	.09*	.05	.05	.11*	–	
15. Uncinate FA	520	.33 (.03)	−.05	.00	.09*	.03	.10*	.08	.04	.08	.04	.05	.09	.10*	.14**	.44***	
16. ILF FA	537	.39 (.04)	−.12**	−.18***	.00	−.06	.02	.03	.01	.00	−.01	−.05	−.08	−.05	−.05	.47***	.38***

* *p* < .05.

** *p* < .01.

*** *p* < .001.

Verbal tests’ scores were used as indicators of two latent factors underpinning verbal ability: Storage and Executive. All five tests (NART, WTAR, and the three letters used in phonemic verbal fluency) were indicators of the Storage latent factor, since all depend on a store of verbal knowledge for successful performance. The three fluency scores were also indicators of the Executive factor. This means that the Executive factor captures shared variance among three phonemic fluency tasks that is *not* shared with the NART and WTAR. This design was motivated by our assumption that, unlike the reading tasks, these tests place additional demands on the ability to flexibly regulate access to verbal representations. The latent factors were fixed to be orthogonal and were identified by fixing their variances to 1. This allowed us to assess the independent contributions of brain structure to each latent factor.

**Fig. 2:**
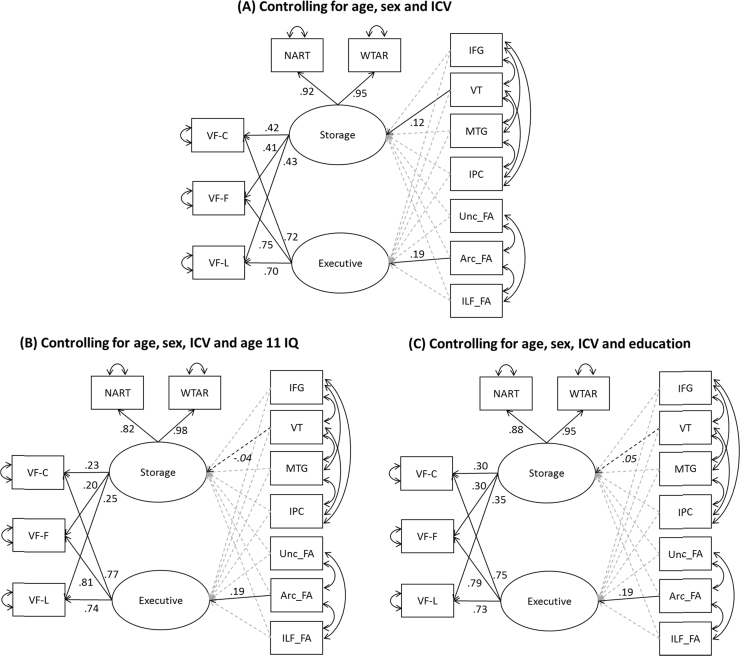
Standardised parameter estimates for structural equation models. Standardised parameter estimates are shown for all significant paths (FDR-adjusted *p* < .05). Paths shown with dashed lines were included in the model but their parameters estimates were not significant (note in particular that the VT-storage paths in Models B and C are not significant).

We tested a series of MIMIC (multiple indicators, multiple causes) models, which are most appropriate for modelling brain-cognition relationships (Kievit et al., 2012, Ritchie et al., 2015). In all cases, volumes of the four GM regions of interest and FA in three tracts of interest were included as predictors of the Storage and Executive factors (see Fig. 2). The models included correlated residuals between GM volumes and correlated residuals between WM FA values. In the first model, variables were adjusted for sex and age, while GM volumes were also adjusted for ICV (as described earlier). The second model was identical except that all variables were additionally regressed on participants’ childhood IQ scores. This model allowed us to assess the degree to which associations in the first model could be accounted for by cognitive level in childhood. As noted earlier, members of the LBC1936 completed the Moray House Test at the age of 11 (Deary et al., 2007), providing a measure of childhood general cognitive ability. Scores on the Moray House Test are strongly correlated with more contemporary intelligence assessment tools, such as the WAIS and Raven's Progressive Matrices (Deary et al., 2010a, Deary et al., 2004). Age 11 IQ scores were available for 525 of the 556 participants who had brain imaging data of suitable quality. In the third model, variables were adjusted for sex, age and ICV and additionally educational attainment (highest level of qualification attained, on a five-point scale). This model allowed us to assess the degree to which associations in the first model could be accounted for time by spent in education.

We assessed model fit using a number of indices and adopted commonly accepted cut-off values for these. We used the following indices (with cut-offs for good fit): Comparative Fit Index (CFI; > .95), Tucker-Lewis Index (TLI; > .95), root mean square error of approximation (RMSEA; < .08) and standardised root mean square residual (SRMSR; < .06). P-values for individual parameter estimates were corrected for multiple comparisons using the false discovery rate (FDR) procedure (Benjamini et al., 2006), which is recommended over the Bonferroni approach for evaluating parameters in structural equation models (Cribbie, 2007).

## Results

Table 2 shows descriptive statistics and correlations between all variables (with brain variables averaged across hemispheres). The two reading measures were strongly correlated with one another and the three fluency measures were also strongly inter-correlated. The two types of task showed moderate correlations with one another (*r* ≈ .4). GM volumes were strongly inter-correlated. This likely reflects their shared covariance with ICV, which was controlled for in all models. FA values were also moderately correlated with one another. There were significant correlations between GM volumes and scores on the NART and WTAR, which may be due in part to the fact that these test scores were also correlated with ICV. VT volume and Arc_FA were also weakly correlated with two of the fluency measures.

Parameter estimates for the first model are shown in Fig. 2 and fit indices in Table 3. The model demonstrated a good fit to the data. Both of the reading tasks loaded very strongly on the Storage latent variable, while the fluency scores showed moderate loadings on this factor and stronger loadings on the Executive factor. Thus, the results indicate that two latent factors of Storage and Executive can be identified in the data. Two brain measurements were significant predictors of the latent verbal factors. VT volume predicted participants’ scores on the Storage factor (β = .12, FDR-adjusted *p* = .04) and Arc_FA predicted scores on the Executive factor (β = .19, FDR-adjusted *p* = .001). The model was able to account for 3.2% of the variance in Storage scores and 4.0% of the variance in Executive scores. Models run on separate left- and right-hemisphere data gave similar results (although the VT-storage parameter failed to reach statistical significance in the left-hemisphere model; see Supplementary Fig. 1).

**Table 3:** Model fit indices for models controlling for age, sex and ICV (A) and additionally, age 11 IQ (B) or educational attainment (C).

	χ^2^	df	*p*-value	CFI	TLI	RMSEA	SRMR	saBIC
Model A	47.3	35	.079	.995	.991	.025	.034	15821
Model B	48.8	35	.061	.993	.987	.027	.036	15508
Model C	47.1	35	.082	.995	.990	.025	.035	16169

CFI = Comparative Fit Index; TLI = Tucker-Lewis Index; RMSEA = Root Mean Square Error of Approximation; SRMR = Standardised Root Mean Square Residual; saBIC = sample-adjusted Bayesian Information Criterion. Note that the chi-square statistic tests for a difference between the actual and modelled data; thus a result of *p* > .05 indicates no significant discrepancy between the fit model and the actual data.

As shown in Table 2, NART and WTAR scores were strongly correlated with IQ at age 11 and with educational attainment, as reported previously (Dykiert and Deary, 2013). These variables were also somewhat predictive of GM volumes, particularly in VT. This raises the possibility that the observed association between VT volume and Storage is confounded by cognitive ability in childhood or by overall educational attainment (which are themselves highly correlated, of course). In the second and third models, we investigated the potential role of these variables in accounting for the associations found in the model. We repeated estimation of the model after regressing all variables on age 11 IQ (Model B) or educational attainment (Model C). Overall fit of the models were good (see Table 3). In both cases, the parameter estimate for the association between Arc FA and the Executive factor were unchanged from Model A (Model B: β = .19, FDR-adjusted *p* = .001; Model C: β = .19, FDR-adjusted *p* = .001). However, the associations between VT volume and the Storage factor were eliminated (Model B: β = −.04, FDR-adjusted *p* = .38; Model C: β = .05, FDR-adjusted *p* = .33). This suggests that the relationship between knowledge representation and VT volume is influenced by experiences earlier in life.

## Discussion

We investigated the neural correlates of two distinct components of verbal ability in over 500 healthy older adults. Based on current models of semantic processing (e.g., Lambon Ralph et al., 2017), we assumed that performance on verbal tasks was underpinned by two underlying abilities: individuals’ ability to store verbal knowledge and their ability to use executive control to flexibly access this information. Structural equation modelling was used to estimate the independent contributions of these two factors to verbal test scores. We found that individual differences in the two factors were predicted by the volume of GM regions in the language/semantic network and by the FA of WM tracts that connect them. Importantly, however, the storage and executive components had distinct neuroanatomical correlates. Volume of the VT cortices, a key site for representation of semantic knowledge, predicted the depth of participants’ knowledge store, while FA of the arcuate fasciculus was predictive of executive skill in regulating access to this information. These associations were small but statistically significant. This is, to our knowledge, the first time such a dissociation has been demonstrated in healthy individuals. At a general level, these data highlight a basic distinction between the neural correlates of information representation and executive control. While representation of knowledge depends on the accumulation of tissue in specialised GM regions, the access and manipulation of this information requires co-ordination across neural networks and thus relies on the integrity of long-range WM pathways. At a more specific level, our results provide insights into the functional architecture of neural networks supporting language processing.

The finding that GM volume in the VT cortices was predictive of participants’ level of verbal knowledge is consistent with a large body of evidence implicating this region in the representation of semantic knowledge (Lambon Ralph, 2014, Patterson et al., 2007). There is a strong association between semantic impairment and dysfunction of this region in the syndrome of semantic dementia (Butler et al., 2009, Mion et al., 2010). The ventral anterior temporal lobe is also reliably activated when healthy individuals perform semantic tasks. fMRI typically provides poor signal in the VT cortices due to their proximity to air-filled sinuses (Ojemann et al., 1997); however, PET studies that are not affected by this problem show activation in this region during verbal semantic processing (Devlin et al., 2000, Spitsyna et al., 2006), as do recent fMRI studies that take steps to improve signal quality (Binney et al., 2010, Halai et al., 2015, Hoffman et al., 2015).

Here, we have shown that cortical volume of the VT cortices is predictive of verbal knowledge in a large sample of older adults. This relationship cannot be attributed to ICV, which was accounted for in our analysis. It is important to note that our ventral region included the entirety of the inferior temporal and fusiform gyri. In the work described above, semantic knowledge representation has been linked specifically with the *anterior* portion of this region. In contrast, the posterior VT lobe plays a central role in visual word recognition (McCandliss et al., 2003), which is also critical for many verbal tasks. The indicators for the Storage factor included visual reading tasks but also verbal fluency tasks that involved no visual presentation. As a consequence, the observed association is likely to reflect lexical-semantic aspects of verbal processing rather than visual word recognition processes.

What is the underlying cause of the association between VT volumes and verbal knowledge in this large sample of healthy older adults? One possibility is that age-related volume reduction in this region leads to deterioration of the knowledge store. In other words, individuals may begin to lose verbal knowledge during the course of healthy ageing in a similar, though much less severe, fashion to patients with semantic dementia. Volume and thickness of the VT cortices do exhibit modest age-related declines, though these are not as pronounced as in many other brain regions (Fjell et al., 2009). Individual differences in the speed and severity of this age-related decline might therefore contribute to the observed association. However, there is also evidence for contribution of earlier life factors to brain structure-function associations in older age and this may provide a more parsimonious account of our findings. It is important to note that receptive vocabulary scores show a gradual improvement across the lifespan which persists into older age (Park et al., 2002, Verhaeghen, 2003). This result, which stands in contrast to marked decline in other cognitive abilities, suggesting that the knowledge store itself does not deteriorate among the population as a whole (although this may not hold true for all individuals, of course). More importantly, we found that the association between VT volume and storage in our participants was entirely eliminated when we controlled for either their IQ at age 11 or their level of educational attainment. This result suggests that our observed effect has its origins much earlier in the lifespan. A similar result was reported by Karama et al. (2014) when investigating brain-wide associations between cortical thickness and later-life IQ in the LBC1936.

We can offer two alternative accounts for this effect (see Karama et al., 2014 for related arguments). One possibility is that genetic influences imbue certain individuals with particularly well-developed temporal lobes, which facilitate the acquisition and retention of verbal semantic knowledge. The Moray House Test used to assess childhood IQ in the LBC1936 includes a number of questions probing verbal reasoning and knowledge of word meanings (Deary et al., 2007). Individuals with more developed knowledge would therefore attain higher IQ scores in childhood (and tend to remain in formal education for longer) and this association between greater knowledge and greater GM volumes could persist into later life. Variations in temporal lobe GM volume have been associated with life-long deficits in verbal semantic abilities. Briscoe et al. (2012) reported a family in which several members from different generations demonstrated a selective, and apparently heritable, deficit in verbal semantic processing. Affected individuals in this family showed reduced VT volumes relative to age-matched controls.

An alternative account views higher childhood intelligence and/or greater time spent in education as confounding factors affecting lifetime exposure to knowledge. Children with higher IQ are likely to remain in education for longer and to work in higher-status occupations (Johnson et al., 2010). This is likely to result in them acquiring a greater quantity of verbal knowledge throughout their life and this increased exposure to knowledge could drive increases in the volumes of the VT cortices throughout the lifespan. There is clear evidence that acquisition of new skills and knowledge during adulthood results in measurable increases in GM volume (Draganski et al., 2006, Engvig et al., 2010, Woollett and Maguire, 2011) and may partly contribute to healthy cognitive ageing (Smart et al., 2014). It is possible, therefore, that large VT volumes are a corollary of a more developed knowledge store, rather than a cause.

The second component of verbal ability we investigated – the ability to apply executive control to access information efficiently – was predicted by FA in the arcuate fasciculus. This association was not explained by childhood IQ and thus may reflect individual differences in the deterioration of this tract during later life (O’Sullivan et al., 2001). The arcuate fasciculus is strongly implicated in language processing as part of the “dorsal” language route and appears to play a critical role in speech production (Fridriksson et al., 2013, Parker et al., 2005, Saur et al., 2008). This view is consistent with the tasks we used to index executive control, which required speeded retrieval and production of words matching a cue. Our findings indicate that this pathway is involved in the flexible retrieval of verbal representations in order to drive speech output. The tract terminates in the posterior part of the ventrolateral prefrontal cortex (Catani and Jones, 2005), a brain region that is strongly associated with selection among competing verbal responses (Badre et al., 2005, Thompson-Schill et al., 1997). Thus, it is possible that the arcuate fasciculus provides the necessary connection between prefrontal executive selection processes and temporal language regions that support performance on this task. Interestingly, the arcuate fasciculus is typically linked with production tasks that tax phonological skills, such as repetition (Hickok and Poeppel, 2007). This accords with the specific demands of the letter fluency task, which requires participants to search for lexical candidates on the basis of their phonological (or more strictly, their orthographic) status and not on their meanings.

The ventral language pathway is more closely associated with semantic processing and access to meaning (Saur et al., 2008). This route includes ILF and uncinate fasciculus, which connect VT and prefrontal regions (Bajada et al., 2015). We found no evidence for a relationship between structure of these tracts and executive regulation of verbal knowledge. This may reflect the demands of the specific tasks we used to index executive processes, which emphasised phonological properties of words rather than their meanings. Other executive tasks that require regulation of word meanings – for example, comprehension of ambiguous words or the detection of meaningful associations (Noonan et al., 2010) – might depend more heavily on the integrity of these pathways. Future studies using a wider range of verbal tasks could provide further insights into these issues, building on our observation that verbal test scores are influenced by multiple underlying components, each with distinct neural bases.

Finally, we note that the strengths of the present study include a much larger sample size than that available in previous studies of individual differences in semantic ability (e.g., de Zubicaray et al., 2011). In addition, the LBC1936 participants span a narrow age range, ensuring that individual differences within the group do not reflect the strongly-confounding effects of age. A further advantage of this particular cohort is the availability of measures of childhood cognitive ability, which allowed us to determine that the association between VT volume and verbal knowledge is related to ability in early life. One potential limitation is that LBC1936 participants have above-average intelligence (Dykiert and Deary, 2013), thus it is possible that we did not sample evenly across the full range of adult ability. We ameliorated this risk through the use of demanding cognitive tests designed to be sensitive to variations in ability in healthy adults. Nevertheless, replications of our findings in other samples is a desirable target for future work.
