# Supplementary material for: Brain grey and white matter predictors of verbal ability traits in older age: The Lothian Birth Cohort 1936
Source: Neuroimage. 2017 Aug 1;156:394–402. doi: 10.1016/j.neuroimage.2017.05.052 (PMC5554782; doi:10.1016/j.neuroimage.2017.05.052)
Supplement: Supplementary file 1 — Supplementary material [file mmc1.docx]

Supplementary Table 1: Correlations between left and right-hemisphere measurements of GM volume and WM fractional anisotropy

|  | **Left-right correlation** |
| --- | --- |
| IFG volume | .72*** |
| VT volume | .79*** |
| MTG volume | .71*** |
| IPC volume | .67*** |
| Arcuate FA | .60*** |
| Uncinate FA | .60*** |
| ILF FA | .47*** |

*** = *p* < 0.001. IFG = inferior frontal gyrus, VT = ventral temporal, MTG = middle temporal gyrus, IPC = inferior parietal cortex, FA = fractional anisotropy, ILF = inferior longitudinal fasciculus.

Supplementary Table 2: Model fit indices for models run on separate left and right-hemisphere data

|  | χ^2^ | df | *p*-value | CFI | TLI | RMSEA | SRMR | saBIC |
| --- | --- | --- | --- | --- | --- | --- | --- | --- |
| Left hemisphere | 44.5 | 35 | 0.131 | 0.996 | 0.993 | 0.022 | 0.025 | 15804 |
| Right hemisphere | 50.8 | 35 | 0.041 | 0.993 | 0.988 | 0.028 | 0.034 | 15851 |

CFI = Comparative Fit Index; TLI = Tucker-Lewis Index; RMSEA = Root Mean Square Error of Approximation; SRMR = Standardised Root Mean Square Residual; saBIC = sample-adjusted Bayesian Information Criterion. Note that the chi-square statistic tests for a difference between the actual and modelled data; thus a result of *p* > 0.05 indicates no significant discrepancy between the fit model and the actual data.

Supplementary Figure 1: Standardised parameter estimates for structural equation models run on separate data from each hemisphere


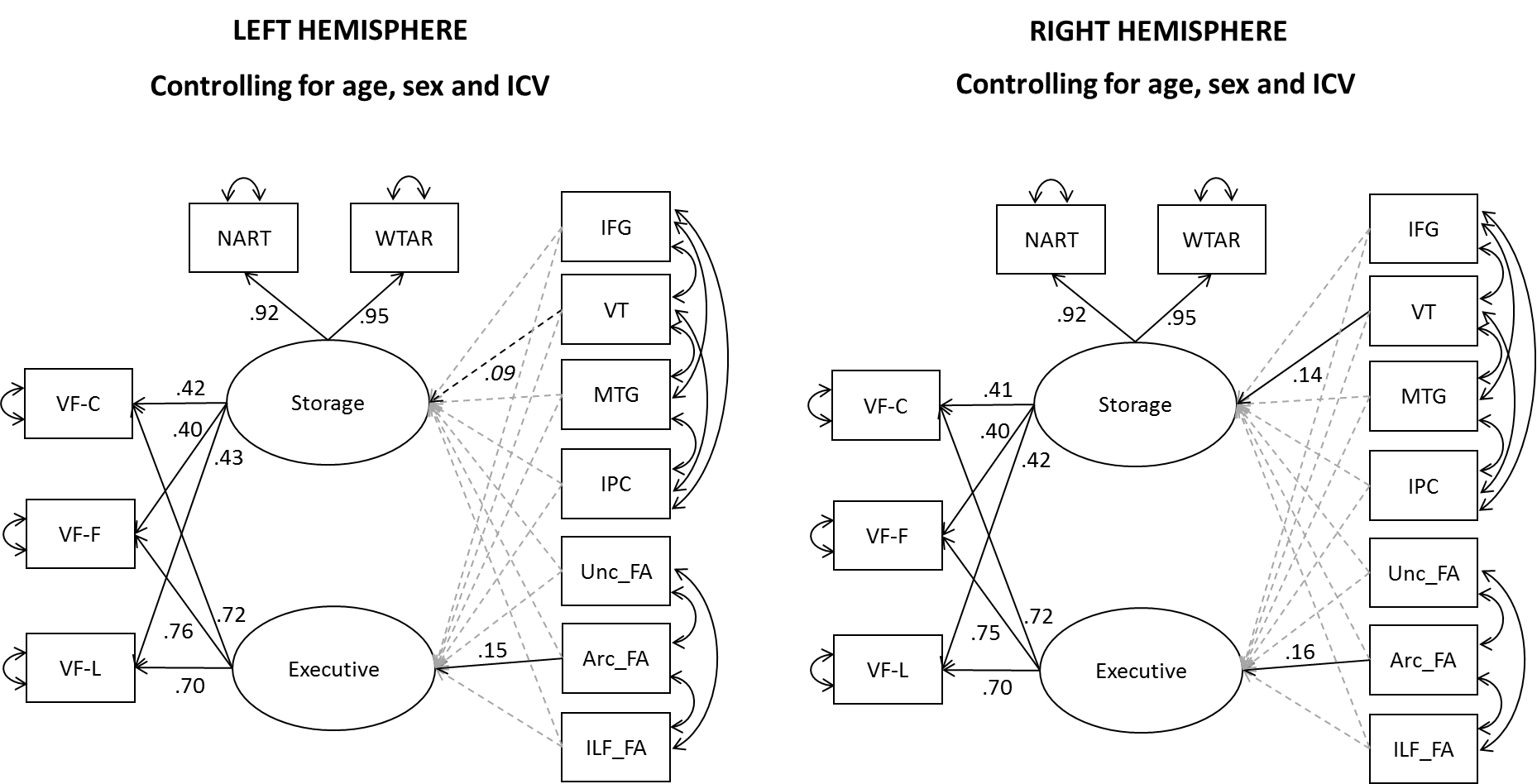


Standardised parameter estimates are shown for all significant paths (FDR-corrected *p* < 0.05). Paths shown with dashed lines were included in the model but their parameters estimates were not significant (note that the VT-storage path in the left-hemisphere model did not reach statistical significance: FDR-adjusted *p* = 0.10).
